# Surgical maneuvers performed on rhinoplasty procedures carried out at an otorhinolaryngology residency program

**DOI:** 10.1016/S1808-8694(15)30986-1

**Published:** 2015-10-19

**Authors:** Lucas Gomes Patrocínio, Paulo Márcio Coelho Carvalho, Hélio Muniz de Souza, Hugo Gonçalves Couto, José Antônio Patrocínio

**Affiliations:** a MD, Otorhinolaryngology Resident - Federal University of Uberlândia; b Full Professor, Head of the Otorhinolaryngology Department - Federal University of Uberlândia. Department of Otorhinolaryngology - Medical School - Federal University of Uberlândia - MG

**Keywords:** graft, incisions, ethnic nose, osteotomy, rhinoplasty

## Abstract

Rhinoplasty is one of the most challenging surgical procedures, due both to the diversity of the techniques and to the difficulty in foreseeing long-term outcomes. Each patient has a different nasal anatomy, dictated by genetic inheritance - race, thus requiring a different technique for each case. The international literature emphasizes the techniques used for the Caucasian nose, which is rarely seen in our region.

**Aim:**

Evaluate and discuss surgical maneuvers used on rhinoplasty procedures performed on local patients at our ENT residency services.

**Materials and Methods:**

We evaluated the operative notes from all patients submitted to rhinoplasty at the Residency Program on Otorhinolaryngology at the Federal University of Uberlândia, from December 2003 to June 2004.

**Results:**

One hundred and sixty-six patients were submitted to rhinoplasty, in which marginal incisions were performed in 118 (71.1%), with the delivery technique performed on the inferior lateral cartilages and some procedures carried out on them (strut, sheen, sutures, etc). Only 45 patients (27.1%) were submitted to basic rhinoplasty and 3 (1.8%) to open rhinoplasty.

**Conclusion:**

Most of our patients demanded additional procedures, and the “basic rhinoplasty”, commonly performed on the Caucasian nose was an exception on our patients.

## INTRODUCTION

Rhinoplasty is a surgical procedure of which technique depends on the anatomy of the nose to be operated upon. Since there are not two noses alike, there are not two identical techniques. The technique varies according to the possible anatomic variations, making it the most challenging of the cosmetic surgeries^1^, ^2^, ^3^.

Since it is a surgery that is highly dependent on the anatomic alterations found, the technique to be used will depend on the type of nose that will be operated. This is influenced by hereditary patterns and, consequentely, by race^4^, ^5^.

International literature emphasizes the Caucasian nose^1^, ^2^, ^3^. In the present study we aim at assessing and discussing the most frequent maneuvers carried out in the patients of our region, located in the Triângulo Mineiro, a place of important race mixing between blacks, whites and Indians (also common to other regions of the country).

## MATERIALS AND METHODS

We have retrospectively assessed the charts of 166 patients who underwent rhinoplasties from December of 2003 to June of 2004, in the Otorhinolaryngology Department of the Medical School of The Federal University of Uberlândia (FAMED-UFU).

From the operation chart of each patient we filled out a form with the following details: access incisions to the nasal bone-cartilage skeleton, maneuvers performed on the inferior lateral and superior cartilages, implants and/or grafts utilized, procedures made to the nasal base, types of osteotomies and number of concomitant septoplasties. All the patients were operated under local anesthesia with intravenous sedation^6^.

The results were plotted on tables and graphs. In percentages, each surgical maneuver was separately analyzed, the need for grafts or incision in relation to the total number of patients operated, thus allowing for a numerical analysis of the approaches carried out in rhinoplasty procedures performed in our department.

## RESULTS

Results are depicted on Table 1, Table 2, Table 3, Table 4, Table 5, Table 6, Table 7, Table 8, Table 9, in relation to the total number of patients operated (166).

**Table 1:** Surgical approach used in the rhinoplasties carried out in the department of Otorhinolaryngology - FAMED-UFU, from December, 2003 to June, 2004.

Approach	N (%)
Delivery	118 (71%)
Closed	45 (27,1%)
Open	03 (1,8%)
Total	166 (100%)

**Table 2:** Types of incisions used in the rhinoplasties carried out in the department of Otorhinolaryngology - FAMED-UFU, from December, 2003 to June, 2004.

Incision	N (%)
Intercartilaginous	147 (88,5%)
Marginal	118 (71,1%)
Intracartilaginous	04 (2,4%)
Transfixating	143 (86,1%)
Paramedian	118 (71,1%)
Transcolumellar	03 (1,8%)

**Table 3:** Maneuver performed on the inferior lateral cartilage in the rhinoplasties carried out in the department of Otorhinolaryngology - FAMED-UFU, from December, 2003 to June, 2004.

Maneuver	N (%)
Resection of the cephalic portion	97 (58,4%)
Domes lateralization	11 (6,6%)
Interdomal suture	75 (45,1%)
Post	40 (24,1%)
Sheen shield cartilaginous graft	36 (21,6%)
Seagull wing	08 (4,8%)

**Table 4:** Procedures used on the nasal septum in the rhinoplasties carried out in the department of Otorhinolaryngology - FAMED-UFU, from December, 2003 to June, 2004.

Procedure	N (%)
Septoplasty	51 (30,7%)
Shortening	64 (38,5%)
Augmentation (Graft)	07 (4,2%)

**Table 5:** Procedures used on the nasal dorsum in the rhinoplasties carried out in the department of Otorhinolaryngology - FAMED-UFU, from December, 2003 to June, 2004.

Procedure	N (%)
Lowering	126 (75,9%)
Graft	11 (6,6%)

**Table 6:** Osteotomies performed in the rhinoplasties carried out in the department of Otorhinolaryngology - FAMED-UFU, from December, 2003 to June, 2004.

Osteotomy	N (%)
Lateral	125 (75,3%)
Medial	14 (8,4%)
Frontal	01 (0,6%)

**Table 7:** Grafts used in the rhinoplasties carried out in the department of Otorhinolaryngology - FAMED-UFU, from December, 2003 to June, 2004.

Graft/Implant	N (%)
Nasal septum cartilage	53 (31,9%)
Pinna cartilage	08 (4,8%)
Dacron®	23 (13,8%)

**Table 8:** Procedure performed on the nasal spine in the rhinoplasties carried out in the department of Otorhinolaryngology - FAMED-UFU, from December, 2003 to June, 2004.

Procedure	N (%)
Removal	11 (6,6%)
Graft	23 (13,8%)

**Table 9:** Procedures performed on the alar base in the rhinoplasties carried out in the department of Otorhinolaryngology - FAMED-UFU, from December, 2003 to June, 2004.

Procedure	N (%)
Alae closure	44 (26,5%)
Stitches	06 (3,6%)

The procedures were performed through three approaches: “delivery” (71.1%), closed (27.1%) or open (1.8%) (Table 1).

As to the incision (s) used to approach the nasal tip, the intercartilaginous was performed in 147 (88.5%) patients; the marginal in 118 (71.1%) and the intracartilaginous in 4 (2.4%) (Table 2).

The most commonly performed maneuvers on the inferior lateral cartilages were the ones used to reduce their volume and enhance nasal tip contour, with a resection of the cephalic portion (58.4%) and interdomal suturing (45.1%), followed by those used to increase support and enhance tip projection, such as the placement of a postgraft (24.1%) and use of a Sheen^7^ shield graft (21.6%) (Table 3).

There was a high incidence of patients who concurrently underwent nasal septoplasty (30,7%), aiming at preserving respiratory function after rhinoplasty. Other maneuvers commonly performed on the nasal septum were its shortening (38.5%) or enlargement, with a cartilaginous graft (4.2%) (Table 4).

On the nasal dorsum, there was a resection of the bone-cartilage bulging in 126 (75.9%) patients and graft placement in 11 (6.6%) (Table 5). Lateral osteotomies were carried out in 125 (75.3%) patients, medial in 14 (8.4%) and frontal in 1 (0.6%) (Table 6).

Among grafts and implants used, the most frequent was septal cartilage (31.9%), followed by Dacronâ (13.8%) and pinna cartilage (4.8%) (Table 7). Most of the patients who underwent procedures on the anterior nasal spine required an augmentation on this region (13.8%) and a lesser number required its removal (6.6%) (Table 8).

The most frequently performed procedure on the wide alar base was its narrowing through resection and suturing (26.5%), followed by an “interalar” suturing (3.6%) (Table 9).

## DISCUSSION

Every rhinoplasty requires access incisions. Proper exposure is as important in rhinoplasties as they are in other types of surgery; therefore, the incisions should be selected as to specific indications. In “Basic Rhinoplasty” the following incisions are performed: transfixating, intercartilaginous and paramedian on the upper lateral cartilage (used to separate the upper lateral cartilage from the septum).

According to Toriumi e Becker^1^, incisions are methods used to give access to bone-cartilaginous structures within the nose, and include the following: transcartilaginous, intercartilaginous, marginal and transcolumellar. We also add the paramedian incision on the upper lateral cartilage to these ones^8^.

Patrocínio et al.^8^ reported that every rhinoplasty requires a careful and precise analysis of what must be corrected. According to Tebbetts^2^, one should use as many incisions as are necessary in order to guarantee ideal exposure and control. The accuracy of the incision may substantially influence its closure quality and subsequent scarring. A precise incision requires tissue stabilization, exposure, planning and an accurate technique.

Incisions for surgical approaches change according to the defect to be corrected. Some authors advocate the transcolumellar external access in 100% of the cases^1^. We used this access in only 1.8% of the cases. In the majority (71.1%) we used marginal incisions, with a “delivery” approach to the inferior lateral cartilages. The low rate of closed rhinoplasties (27.1%) shows the high incidence of deformities on the inferior lateral cartilages that need surgical correction^4^, ^5^.

Trans/intracartilaginous access in primary rhinoplasties, in which all we need is to reduce the tip volume, is carried out through a 4 to 6mm incision caudal to the cephalic border of the lateral crura. In this procedure, what matters is what we leave behind, and not what we take from the cartilage. This access allows for tip reduction and refining, without the risk of altering the nasal valve^3^.

The marginal incision is the one in which we obtain a doubly pedicled flap (Figure 2). For the “delivery” approach, we perform both the marginal and the intercartilaginous incisions^8^. Almost all the procedures carried out by open rhinoplasty can be performed through this access, without leaving a columellae scar. Open rhinoplasty was used in only 3 of our cases. The advantage of this procedure is direct visualization of the alar cartilages, making it easier to place sutures and grafts, besides facilitating teaching. It is well indicated for fissure noses and tertiary or quaternary rhinoplasties, those with important pinching and assymetries^4^, ^5^. Most secondary rhinoplasties are lesser surgeries than the first ones, and are used to correct subtle defects and, therefore, do not require the open procedure.

As to the actions to be performed on the lower lateral cartilages, the cephalic portion was resected in order to better define the tip in 58.4% of the patients. Interdomal suturing, post placement, Sheen shield cartilaginous graft placement, domes lateralization and “seagull wing” type of graft were performed in 45.1%, 24.1%, 21.6%, 6.6% and 4.8% patients, respectively. According to Sheen^7^, the four points that define nasal tip are: supratip breakpoint, right “domes”, left “domes” and lobe-columellar junction; and the abovementioned maneuvers aimed at acting on these points. Maybe it is because we are otorhinolaryngologists that we had so many nasal septoplasties (30.7%), allowing not only a functional improvement, but also the supply of grafting cartilage^9^. In only 4.2% of the cases a graft was placed on the causal septum because of a small naso-labial angle.

Lateral osteotomy was used in 75.3% of the cases, and this may be explained by the large number of noses with broad bone base in our region^4^, ^5^. For men we used the 3mm osteotome and for women, the 2mm, with an access that is lateral and superior to the head of the inferior nasal conchae, with a dotted-line type of fracture in an ascending fashion to the naso-maxillary angle.

Nasal septum cartilage grafting was used in 31.9% of the patients, pinna cartilage in 4.8% and Dacronâ in 13.8%. Septal cartilage was used mainly to make the post and the Sheen shield grafts, and the pinna was used for dorsum grafts^10^; “seagull wing” and Dacronâ were used for the anterior nasal spine. The latter, since it is a soft material, it is not used for support, but as filling substance only, and it is easy to obtain in our settings, because since all we need is a small quantity, we use the sterilized remains of what is used by the Vascular Surgery Department. There are other types of implants described in the literature, such as Gore-texâ, Supramidâ, Proplastâ and hydroxyapatite^11^, ^12^, ^13^.

Access to the anterior nasal spine, for resection in 6.6% of the cases or grafting in 13.8%, was carried out by the columellar transfixating incision and pouch creation. In these cases, the most frequently used material was Dacronâ (soaked in Clindamycin), placed at the end of the procedure and through a very well closed incision.

It is preferable to use cartilage; however, due to a great need for augmentation, we use Dacronâ in order to avoid a new incision for graft removal, in this case, from the other ear^11^, ^12^.

As to the nasal base, we approached it in 30.1% of the patients, either by base resection with suturing^4^, ^5^, ^13^ in 26.5% and by interalar stitches in 3.6%, which is indicated when there is a subtle increase in the interalar distance. According to Daniel^3^, the lateral alar incision is not usually necessary, and it always leaves a scar behind. We did not use this type of incision on our patients.

## CONCLUSION

In the present study, we carried out maneuvers that complement the ones used in basic rhinoplasty. Most of the patients underwent some kind of procedure on their inferior lateral cartilages, and a large number of them underwent lateral osteotomy, nasal alae closure, graft and/or implants placement and other maneuvers. Basic rhinoplasty, usually carried out in Caucasians, was an exception in our settings.
